# Exploring the therapeutic potential of garlic in alcoholic liver disease: a network pharmacology and experimental validation study

**DOI:** 10.1186/s12263-024-00748-3

**Published:** 2024-07-23

**Authors:** Siqi Gao, Tingting Gao, Lizheng Li, Shule Wang, Jie Hu, Ruijing Zhang, Yun Zhou, Honglin Dong

**Affiliations:** 1 Department of Vascular Surgery, The Second Hospital of Shanxi Medical University, Taiyuan, China; 2 Department of Nephrology, The Second Hospital of Shanxi Medical University, Taiyuan, China; 3 Shanxi Province Integrated Traditional and Western Medicine Hospital, Taiyuan, China

**Keywords:** Alcoholic liver disease, Garlic, Network pharmacology, Molecular docking, Alcohol dehydrogenases

## Abstract

**Objective:**

Employing network pharmacology and molecular docking, the study predicts the active compounds in garlic and elucidates their mechanism in inhibiting the development of alcoholic liver disease (ALD). ALD is a global chronic liver disease with potential for hepatocellular carcinoma progression.

**Methods:**

The main active ingredients and targets of garlic were identified through screening the TCMSP, TCM-ID, and ETCM databases. ALD disease targets were sourced from DisGeNET, GeneCards, and DiGSeE databases, and intervention targets for garlic were determined through intersections. Protein interaction networks were constructed using the STRING platform, and GO and KEGG pathway enrichment analyses were performed with R software. The garlic component-disease-target network was established using Cytoscape software. Validation of active ingredients against core targets was conducted through molecular docking simulations using AutoDock Vina software. Expression validation of core targets was carried out using human sequencing data of ALD obtained from the GEO database.

**Results:**

Integration of garlic drug targets with ALD disease targets identified 83 target genes. Validation through an alcohol-induced ALD mouse model supported certain network pharmacology findings, suggesting that garlic may impede disease progression by mitigating the inflammatory response and promoting ethanol metabolism.

**Conclusion:**

This study provides insights into the potential therapeutic mechanisms of garlic in inhibiting ALD development. The identified active ingredients offer promising avenues for further investigation and development of treatments for ALD, emphasizing the importance of botanical remedies in liver disease management.

**Supplementary Information:**

The online version contains supplementary material available at 10.1186/s12263-024-00748-3.

## Introduction

About 2 million people worldwide die from liver disease each year, and alcoholic liver disease (ALD) is the most common chronic liver disease in the world [1]. ALD is caused by chronic alcohol consumption above a certain daily allowance and varies significantly between individuals [2]. Epidemiological studies have shown that alcohol is a major cause of advanced liver disease in Europe, the United States and China [3, 4]. ALD follows a recognized pattern of disease progression that can progress from early alcoholic fatty liver to alcoholic steatohepatitis, characterized by liver inflammation, hepatocellular damage, hepatocellular ballooning, and eventually progressive fibrosis and cirrhosis and, in some cases, hepatocellular carcinoma [5–7]. The pathogenesis of ALD includes hepatic steatosis, oxidative stress, acetaldehyde-mediated toxic effects, and cytokine and chemokine-induced inflammation [8, 9]. Despite the great harm, progress in ALD research has been slow, and current improvements in the etiology of ALD have focused on less efficient interventions to reduce alcohol consumption [10]. In symptomatic treatment, adverse drug reactions, especially severe toxicity, lead to limited treatment of patients with ALD [11, 12]. With the boom of herbal remedies for human diseases in recent decades, a series of studies have focused on the beneficial effects of garlic on ALD [3].

Garlic (Allium sativum, DASUAN) is an aromatic herb whose potential medicinal value dates back thousands of years and is one of the world’s most popular herbs [13]. The active ingredients of garlic include enzymes, sulfur-containing compounds and compounds produced enzymatically from alliin [14]. Studies have shown that garlic plays a protective role in inflammatory responses, abnormal ethanol metabolism, impaired lipid function metabolism and cellular peroxidative damage [15–18]. However, the mechanism underlying garlic-mediated liver protection remains unclear, necessitating further studies to determine its protective effect on ALD and to elucidate the potential mechanism. Consequently, we established an alcoholic liver injury mouse model to assess the impact of administering garlic on alcohol-induced liver injury, providing a foundation for future research.

Hopkins proposed the research method of network pharmacology in 2007 [19]. It relies on systems biology theory and entails constructing biological networks using high-throughput omics data, diverse databases, and literature, employing computer science and technology as primary tools. This method enables a comprehensive analysis of the efficacy, toxicity, bioavailability, and mechanism of action of new drugs [20]. Molecular docking is one of the most commonly used classic methods in receptor-based virtual screening. It is a computational screening process based on the protein structures corresponding to diseases [21]. Molecular docking can score the binding interactions between active compounds and their targets, virtually screening for potentially effective components in traditional Chinese medicine formulations or herbs. This technique is widely used in studying the mechanisms of traditional Chinese medicine and evaluating its active ingredients [22, 23]. By performing molecular docking on the compounds and targets identified through network pharmacology, the binding stability between active ingredients and their targets can be further validated. The combined use of these two methodologies is an effective means of investigating the core active components and their targets in medicinal compounds [24].

**Fig. 1:**
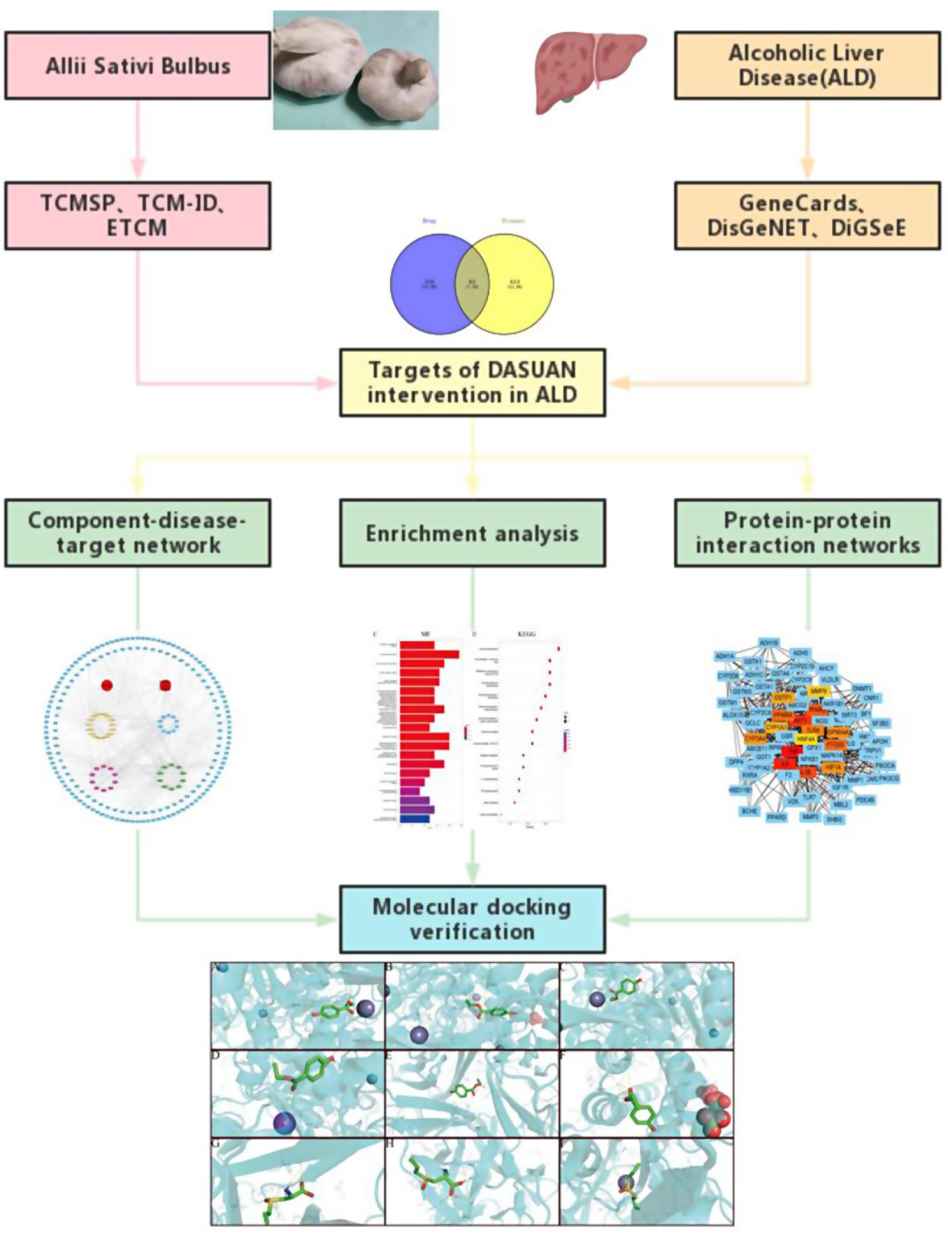
A flow chart illustrated the idea of the project. The photos of garlic were taken recently by the author and are copyright free

In this study, the main components of action and targets of intervention of garlic were explored through network pharmacology and molecular docking techniques. The workflow diagram is shown in Fig. 1.

## Materials and methods

### Screening of the main components and targets of garlic

The main active ingredients of garlic were obtained from the Traditional Chinese Medicine Systems Pharmacology Database and Analysis Platform (TCMSP, https://old.tcmsp-e.com/index.php). Oral bioavailability > 30% is the screening criterion for active ingredients. At the same time, through the information database of traditional Chinese medicine (TCM-ID, https://www.bidd.group/TCMID/) and China encyclopedia (ETCM) of traditional Chinese medicine database “DASUAN” input keywords search, get active ingredients of garlic and key targets [25]. Together, these three databases provide the basis for subsequent research.

### Therapeutic targets for ALD

The potential targets of ALD were obtained from DisGeNET [26], GeneCards [27] and DiGSeE [28] databases. The search subject terms in the DisGeNET database were “Alcoholic liver diseases”, “Hepatitis, Alcoholic”, “Liver Cirrhosis, Alcoholic”, “Chronic Alcoholic Hepatitis”, “Acute alcoholic liver disease”, “Fatty Liver, Alcoholic” and “Alcoholic Steatohepatitis”, with score GDA (Gene-Disease Association Score) > 0.01. The search terms in the GeneCards database were “Alcoholic liver disease”. The search terms used in the DiGSeE database were “Alcoholic liver disease,” with a relevance score of evidence sentences greater than 0.1. The three databases were concatenated as the targets for ALD.

### Garlic as a potential target for intervention in ALD

Using Venny 2.1.0 online mapping software (https://bioinfogp.cnb.csic.es/tools/venny/), the targets of garlic action were intersected with the targets of alcoholic hepatitis.

### Protein-protein interaction network construction and core target screening

Eighty-three target genes were imported into the String database [29] to construct the Protein-Protein Interaction Network (PPI). The data files were exported in TSV format and imported into Cytoscape 3.9.1 software [30], and the PPI network was analyzed using CytoHubba plugin. The top 15 core genes were obtained based on the degree, maximal clique centrality (MCC), maximum Neighborhood Component (MNC), edge-percolated component (EPC) and closeness algorithms. The intersection was performed using Draw Venn Diagram online mapping software (https://bioinformatics.psb.ugent.be/webtools/Venn/) to obtain 10 core targets. Because of its high confidence, the MCC algorithm is used to rank the core targets in descending order.

### Component-disease-target interaction network construction

The previously screened garlic intervention targets were intersected with the potential therapeutic targets for ALD. The major components identified from this intersection were then searched in the STRING database to retrieve the corresponding GO terms and KEGG pathways. This information was subsequently imported into Cytoscape software. The component-disease-target interaction network was obtained. The connecting lines between nodes represent the existence of relationships between them.

### GO and KEGG pathway enrichment analysis

The ClusterProfiler package in R was used for Gene Ontology (GO) and Kyoto Encyclopedia of Genes and Genomes (KEGG) pathway enrichment analysis, with a threshold of *P* < 0.05 for statistical significance. The 83 potential targets were run through the program, and the top 20 GO topics for molecular function (MF), cellular component (CC) and biological process (BP) and the top 15 KEGG pathways were selected for further analysis.

### Molecular docking and heat map analysis

The major active components of garlic were used as ligands, and important genes of interest were selected one by one from the PPI network as receptors for molecular docking. The 3D structures of the active ingredients of garlic were obtained from the TCMSP database, the receptor protein structures using UniProt and PDB databases, and performed dehydration and de-residue operations using PyMol software. Then they were imported into AutoDockTools 1.5.7 software [31] to be converted into PDBQT format. Molecular docking was then performed using AutoDock Vina software [32], and the results were visualized and analyzed using PyMol software [33].

The affinity of molecular docking was expressed as molecular binding energy, and less than − 4.2 kcal/mol was considered to have some binding strength. The main active ingredients and target genes were imported into MeV 4.9.0 software for thermogram analysis.

### Expression of key genes in clinical samples of ALD

The GSE28619 dataset [34] was found in the GEO database [35], which includes liver tissue from 7 healthy individuals and 15 patients with alcoholic hepatitis. The Xiantao academic database was used for analyzing disease-related gene expression through its online platform (https://www.xiantao.love/).

### Animal grouping and establishment of alcoholic liver disease model

Sixteen male C57BL/6J mice (8 weeks old) were housed in plastic cages with four mice per cage, maintained at 23 °C with 60% relative humidity for a 12-hour light/dark cycle. The mice had unrestricted access to a liquid diet. Before the animal experiment, 24 mice were randomly divided into four groups (*n* = 6). Ethical approval for this experiment was obtained from the Second Hospital of Shanxi Medical University (No. DW2023050). The liquid ethanol feed formula was provided by Nanjing Junke biological. The ALD group received an L10016 alcohol liquid diet (132.18 g diet, 50 g absolute ethanol, water to 1 L; 1000 kcal/L) for 4 weeks. The C57BL/6 group was fed an L10015 liquid diet (132.18 g diet, 89.6 g maltodextrin, equal volume of water to 1 L; 1000 kcal/L) for 4 weeks. The ALD + Allicin group was given an L10016 ethanol liquid diet (132.18 g diet, 50 g absolute ethanol, water to 1 L; 1000 kcal/L) for 4 weeks, with daily allicin (10 mg/kg) administered by gavage. The C57BL/6 + Allicin group (negative control group) was fed a normal diet instead of a liquid diet for 4 weeks, with daily allicin (10 mg/kg) given by gavage. C57BL/6J mice were euthanized by intraperitoneal injection of nembutal (60 mg/kg). Blood and liver samples were collected. The liver tissue was washed with saline, and tissue from the second large lobe of the liver was isolated and fixed in 10% formalin for histopathological examination. The remaining liver samples were stored at -80 °C.

### Histological analysis of tissues

Mice liver tissues were first rinsed with cold PBS and subsequently fixed in 4% paraformaldehyde for more than 12 h. Paraffin-embedded sections were then stained with hematoxylin and eosin for histological examination. Additionally, frozen sections of the liver were fixed with paraformaldehyde and subjected to oil red O staining. The observed histological changes were examined using an upright microscope.

### Determination of serum inflammatory factors

The serum of mice was detected by ELISA, and the detection indicators and kits were as follows: TLR4 (ZCI Bio, ZC-37,841), IL-1 (ZCI Bio, ZC-37,961), IL-6 (ZCI Bio, ZC-37,988), and TNF-α (ZCI Bio, ZC-39,024). Please strictly follow the manufacturer’s instructions.

### Immunohistochemical staining

The paraffin sections underwent dewaxing and antigen repair processing, followed by incubation with goat serum for 30 min at room temperature. Primary antibodies, including TLR4 (Servicebio, GB11519), IL-1β (Servicebio, GB11113), IL-6 (Servicebio, GB11117), and TNF-α (Servicebio, GB11188), were added drop by drop at appropriate concentrations and incubated overnight at 4 °C in a wet chamber. The following day, a secondary antibody of the same species as the primary antibody was applied to the tissue. After a 1-hour incubation at room temperature, the nuclei were stained with hematoxylin. Finally, the slices were dehydrated, sealed, and the results were interpreted using a white light microscope.

### Immunofluorescence staining

Paraffin sections underwent deparaffinization, followed by antigen repair and serum blocking. Subsequently, primary antibodies were added and incubated overnight at 4 °C in a wet box. The following day, corresponding Cy3-labeled secondary antibodies were applied, incubated, and washed. Nuclei were preserved using DAPI staining solution, and sealing was achieved with an anti-fluorescence quenching blocker. Finally, image acquisition was performed, and Image J was utilized for image analysis. The antibodies employed in this experiment include ADH1A/ADH1B/ADH1C (ABclonal, A18581) and ADH5 (ABclonal, A2041).

### Western blot analysis

Mice liver tissues underwent processing in enhanced RIPA lysate and thorough pulverization using an ultrasonic tissue morcellator. Quantification of proteins was carried out using the BCA Protein concentration assay kit pairs following the manufacturer’s instructions. Subsequently, total proteins underwent separation through SDS-PAGE gel electrophoresis. The separated proteins were then transferred to 0.45 μm PVDF membranes. Blocking was performed with TBST buffer containing 5% skim milk powder at room temperature. Subsequently, proteins were incubated with primary antibodies overnight at 4 °C. Subsequently, they were incubated with appropriate secondary antibodies for 120 min at room temperature. Finally, protein signals were developed using ECL luminescence solution in the ChemiDoc system. Protein expression levels were analyzed using Image Lab software. A reference protein (GAPDH, Servicebio, GB15002) was employed to normalize the target proteins, including TLR4 (Servicebio, GB11519), IL-1β (Servicebio, GB11113), IL-6 (Servicebio, GB11117), and TNF-α (Servicebio, GB11188).

### Statistical analyses

The data of this experiment were expressed as mean ± standard deviation. Statistical differences among groups were analyzed by one-way ANOVA or t-test. All statistical analysis data and graphs were obtained using GraphPad Prism 10.1.0 software. *P* < 0.05 was considered to be statistically significant.

## Results

### Active ingredients of garlic and targets of intervention

**Fig. 2:**
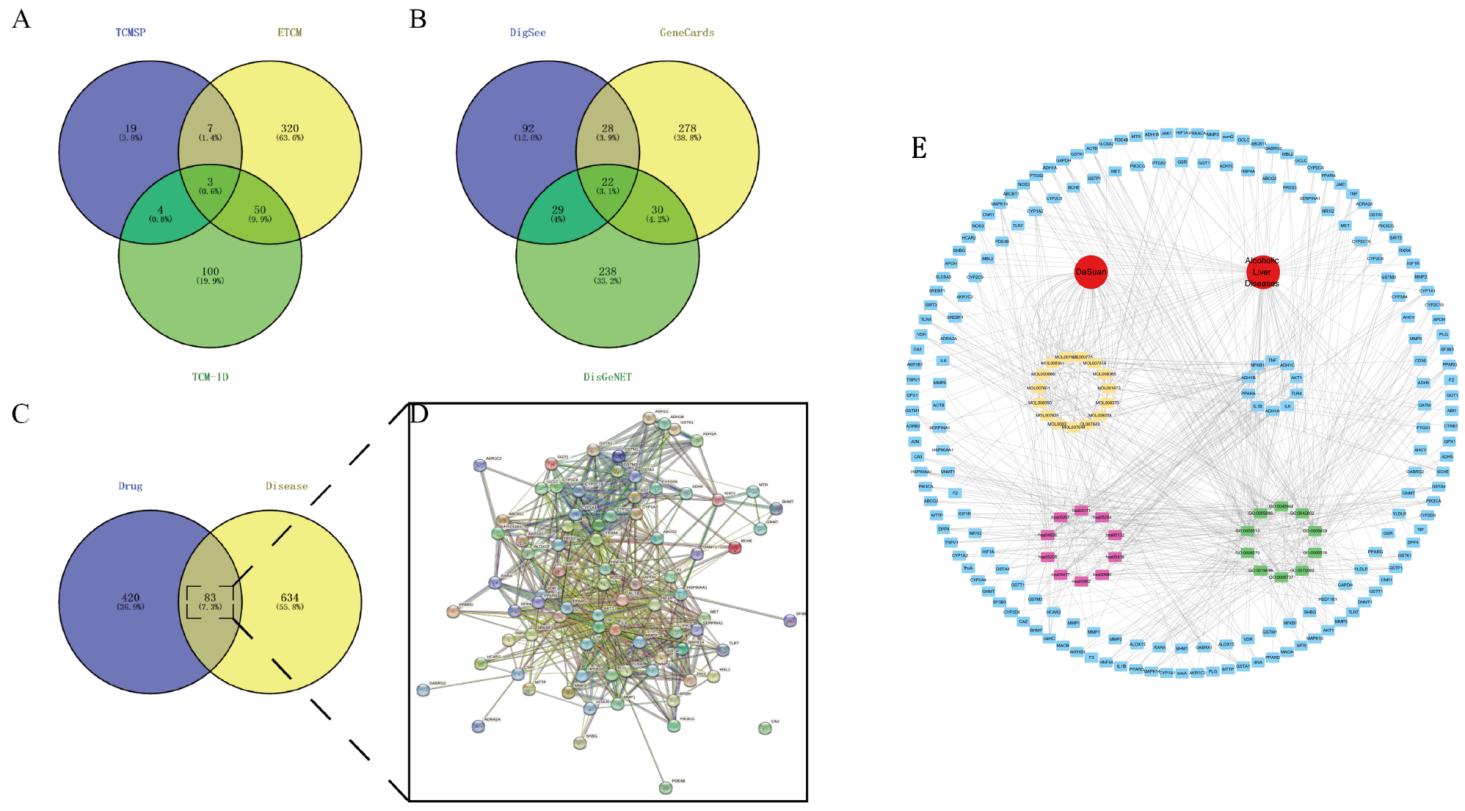
(**A**) The intervention targets of garlic were obtained from three databases, and the total targets were obtained by taking the concatenation. (**B**) Potential therapeutic targets for ALD were obtained from three databases, and the total targets were obtained by taking the concurrent set. (**C**) The total intervention targets of garlic were intersected with the total potential therapeutic targets of ALD. The potential targets of garlic for the treatment of ALD were obtained. (**D**) PPI network of potential targets. (**E**) A network of garlic interventions for ALD. The yellow marker is the main active ingredient of garlic. The blue markers are potential targets of action. The pink marker is the KEGG pathway. The green markers are GO analysis topics

A search was conducted in the TCMSP database, considering compounds with oral bioavailability (OB) greater than 30% and excluding active ingredients with low specificity. This yielded a total of 26 active ingredients, as detailed in Table 1. In addition, the intervention targets of garlic were collected through multiple databases, and we found a total of 503 potential targets for garlic (Fig. 2A).

**Table 1:** The main active ingredients of “DASUAN”

Mol ID	Molecule Name	MW	OB(%)
MOL001873	Sobrol A	166.19	64.98
MOL004046	Dimethyl tetrasulfide	158.36	51.34
MOL000666	hexanal	100.18	55.71
MOL007601	(+)-L-Alliin	177.25	86.68
MOL007619	Methyl allyl sulfide	88.19	70.09
MOL007643	methylallyl disulphide	120.26	73.64
MOL007644	Methyldithio-1-propene	120.26	73.26
MOL008352	3-(isoamylthio)prop-1-ene	144.31	60.6
MOL008354	Allicin	162.3	78.41
MOL008358	Oil garlic	114.23	74.81
MOL008359	DATS	210.44	49.42
MOL008364	Dimethylthiourea	104.2	72.92
MOL008365	Propyl n-butyl disulfide	164.37	62.95
MOL008368	2,2-Dimethoxyethylamine	105.16	54.3
MOL008369	3-Butenoic acid	86.1	71.76
MOL008370	2,5-Dimethylthiophene	112.21	49.64
MOL008372	2-ethyl-1,3-dithiane	148.32	44.35
MOL008373	2-ISOPROPYL-1,3-DIOXOLANE	116.18	109.06
MOL008374	3,4-Dimethylthiophene	112.21	84.37
MOL007621	DMDS	94.22	39.27
MOL007627	diAllS2	146.3	49.28
MOL008356	Benzaldoxime	121.15	32.46
MOL000775	EEE	88.12	45.02
MOL008350	3-Ethenyl-1,2-dithia-cyclohex-5-ene	144.28	44.6
MOL008351	3-Ethylthiophene	112.21	40.37
MOL008361	3-METHYL-2-THIABUTANE	90.21	31.49

### Potential therapeutic targets for ALD

717 major targets for ALD were obtained from the DisGeNET, GeneCards and DiGSeE databases (Fig. 2B). Then the targets of garlic were intersected with the targets related to ALD to obtain 83 potential therapeutic targets (Fig. 2C).

### Garlic active ingredient-ALD-target interaction network

The 83 potential therapeutic targets were imported into the String database to obtain the PPI network (Fig. 2D), and the GO themes and KEGG pathways were obtained. Then we imported the active ingredients, potential therapeutic targets, GO themes and signaling pathways into Cytoscape software to obtain the ingredient-disease-target interactions network (Fig. 2E). The blue color is the target, the yellow color is the main active ingredient of garlic, the pink color is the top ten signaling pathways, and the green color is the top ten GO topics. The red circles are the subject terms of garlic and ALD, respectively.

### GO and KEGG pathway enrichment analysis

**Fig. 3:**
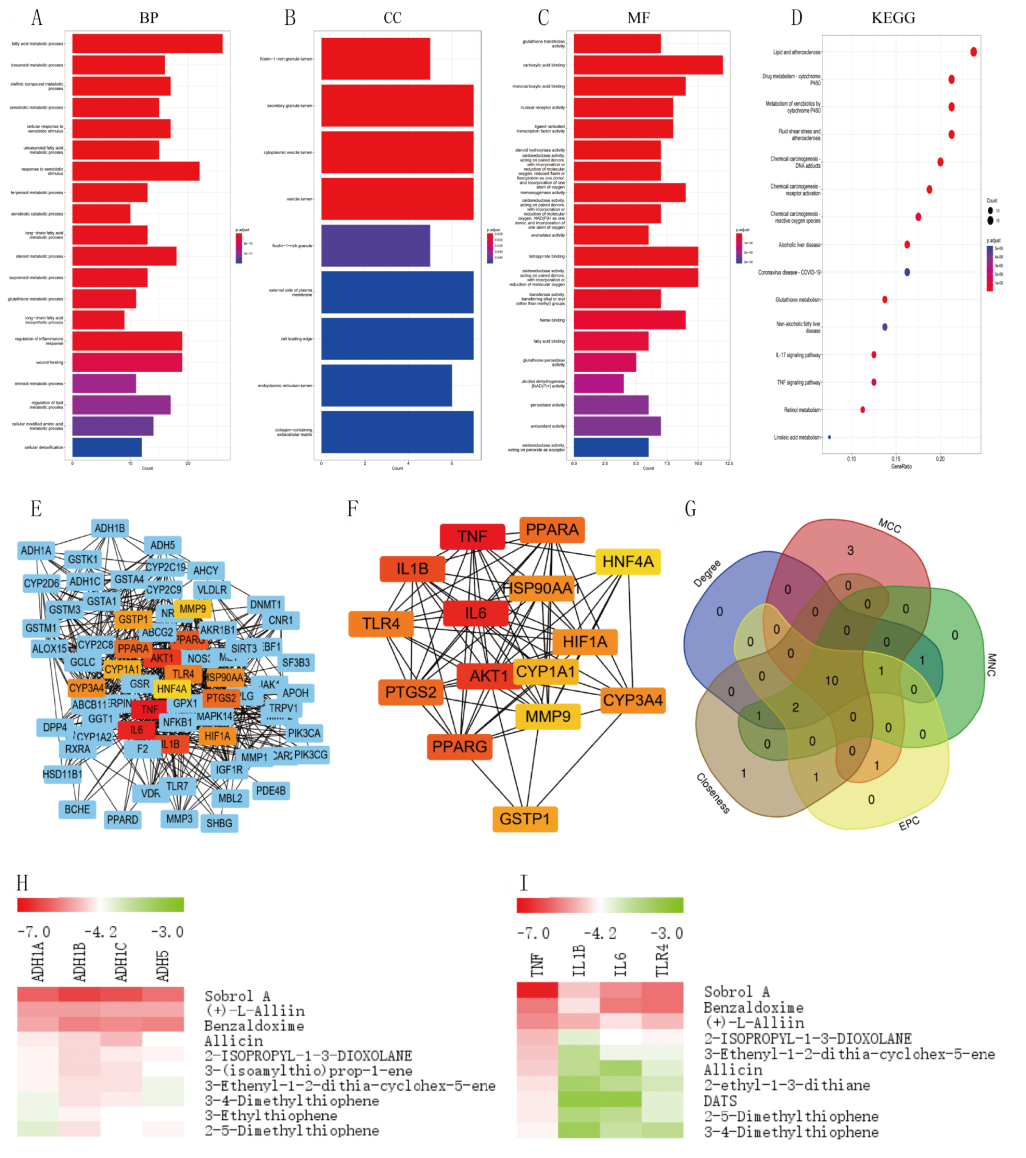
Display of enrichment analysis part results. (**A**-**C**) Based on Go enrichment analysis, functions in which garlic targets may be involved in ALD, including biological process, cellular component and molecular function. (**D**) KEGG analysis results showed pivotal signaling pathways of garlic intervention in ALD. The size of the bubble represents the number of genes, and the color of the bubble represents the size of the p-value. The darker the color, the smaller the P-value. (**E**) PPI network of 83 targets. (**F**) Among them, the top 15 significant genes obtained according to the Degree algorithm of CytoHubba plugin are shown in red. (**G**) The top 15 core genes were obtained based on Degree, MCC, MNC, EPC and Closeness algorithms. The intersection was performed using Draw Venn Diagram online mapping software to obtain the 10 core targets. (**H**) Thermogram analysis of the molecular binding energy of the active components of garlic to ADH. (**I**) Thermogram analysis of molecular binding energies of active components of garlic to inflammatory factors and TLR4

GO and KEGG pathway enrichment analysis was performed on 83 cross-targets using RStudio, with a threshold of *P* < 0.05 for statistical significance. The functions of the genes were enriched into three parts: MF, CC and BP. 1034 BPs, 9 CCs and 87 MFs were obtained, with the first 15 significant themes for each module as in Fig. 2E. The GO-BPs of the targets mainly consisted of fatty acid metabolic process, icosanoid metabolic process, olefinic compound metabolic process, xenobiotic metabolic process and cellular response to xenobiotic stimulus (Fig. 3A).

GO-CC consists of ficolin-1-rich granule lumen, secretory granule lumen, cytoplasmic vesicle lumen, and vesicle lumen (Fig. 3B). GO-MF includes alcohol dehydrogenase NAD(P) + activity, glutathione transferase activity, carboxylic acid binding and monocarboxylic acid binding, etc. (Fig. 3C).

In addition, the potential targets are mainly enriched in 107 KEGG pathways such as Lipid and atherosclerosis, Drug metabolism-cytochrome P450, Metabolism of xenobiotics by cytochrome P450, Chemical carcinogenesis-DNA adducts, ALD and Coronavirus disease - COVID-19 (Fig. 3D). Each signalling pathway consists of a different target, with our focus on the ALD-related signalling pathway (Supplementary Fig. 1).

### Analysis of protein-protein interaction networks

PPI network analysis **(**Figs. 2D and 3E) was performed on 83 target genes using the String database with the following screening criteria: the species was Homo sapiens and the minimum required interaction score was 0.4. The core targets were screened further using Cytoscape software. We employed Degree, MCC, MNC, EPC, and close algorithms from the CytoHubba plugin to identify the top 15 key genes. (Table 2; Fig. 3F). These five groups of genes were then intersected to obtain 10 core targets, ordered by the MCC algorithm as IL6, TNF, AKT1, IL1B, HIF1A, PTGS2, TLR4, PPARG, HSP90AA1 and PPARA (Table 3; Fig. 3G). With reference to the signalling pathways associated with ALD, we suggest that these 10 genes can be the core genes of garlic for the treatment of ALD.

**Table 2:** The top 15 genes of cytoHubba screening

Rank	Rank methods in CytoHubba				
	Degree	MCC	MNC	EPC	Closeness
1	TNF	IL6	TNF	TNF	TNF
2	IL6	TNF	IL6	IL6	IL6
3	AKT1	AKT1	AKT1	AKT1	AKT1
4	IL1B	IL1B	IL1B	IL1B	IL1B
5	PPARG	HIF1A	PPARG	PTGS2	PPARG
6	PPARA	PTGS2	PPARA	PPARA	PTGS2
7	PTGS2	MMP9	PTGS2	PPARG	PPARA
8	TLR4	TLR4	TLR4	TLR4	HSP90AA1
9	CYP3A4	NOS3	CYP3A4	HIF1A	CYP3A4
10	HIF1A	PPARG	HIF1A	HSP90AA1	TLR4
11	HSP90AA1	HSP90AA1	GSTP1	MMP9	HIF1A
12	GSTP1	MMP2	HSP90AA1	NOS3	GSTP1
13	CYP1A1	MAPK14	CYP1A1	CYP1A1	CYP1A1
14	MMP9	PLG	MMP9	CYP3A4	GSR
15	HNF4A	PPARA	HNF4A	GSR	GPX1

**Table 3:** Information on the 10 key targets

Entry ID	Gene	Protein
P05231	IL6	Interleukin-6
P01375	TNF	Tumor necrosis factor
P31749	AKT1	RAC-alpha serine/threonine-protein kinase
P01584	IL1B	Interleukin-1 beta
Q16665	HIF1A	Hypoxia-inducible factor 1-alpha
P35354	PTGS2	Prostaglandin G/H synthase 2
O00206	TLR4	Toll-like receptor 4
P37231	PPARG	Peroxisome proliferator-activated receptor gamma
P07900	HSP90AA1	Heat shock protein HSP 90-alpha
Q07869	PPARA	Peroxisome proliferator-activated receptor alpha

### Molecular docking verification

The more stable the binding conformation of the receptor and ligand, the lower the molecular binding energy, and we consider a binding activity of less than − 4.2 kcal/mol to be meaningful. We performed molecular docking of the core targets of inflammatory factors IL6, IL1B and TNF, receptor TLR4 and alcohol dehydrogenases ADH1A, ADH1B, ADH1C and ADH5 with the main active ingredients one by one to obtain the active ingredients of garlic. The results of the molecular binding energies are shown as a heat map in Fig. 3H, I, with specific information in Table 4.

**Table 4:** Molecular docking results of core targets and active components

Target	PDB ID	Component	Docking score (kcal/mol)
IL6	1IL6	Sobrol A	-5.4
		Benzaldoxime	-5.6
		(+)-L-Alliin	-4.5
TNF	1A8M	Sobrol A	-6.7
		Benzaldoxime	-5.6
		(+)-L-Alliin	-5.4
		2-ISOPROPYL-1-3-DIOXOLANE	-4.9
		3-Ethenyl-1-2-dithia-cyclohex-5-ene	-4.8
IL1B	1IOB	Sobrol A	-4.8
		Benzaldoxime	-4.5
		(+)-L-Alliin	-5.0
TLR4	3FXI	Sobrol A	-5.7
		Benzaldoxime	-5.7
		(+)-L-Alliin	-4.9
ADH1A	1HSO	Sobrol A	-5.9
		(+)-L-Alliin	-5.2
		Benzaldoxime	-5.1
		Allicin	-4.4
ADH1B	3HUD	Sobrol A	-6.3
		(+)-L-Alliin	-5.2
		Benzaldoxime	-5.5
		Allicin	-4.6
ADH1C	1HT0	Sobrol A	-6.1
		(+)-L-Alliin	-5.1
		Benzaldoxime	-5.4
		Allicin	-4.9
ADH5	3QJ5	Sobrol A	-5.7
		(+)-L-Alliin	-5.1
		Benzaldoxime	-5.5
		Allicin	-4.2

**Fig. 4:**
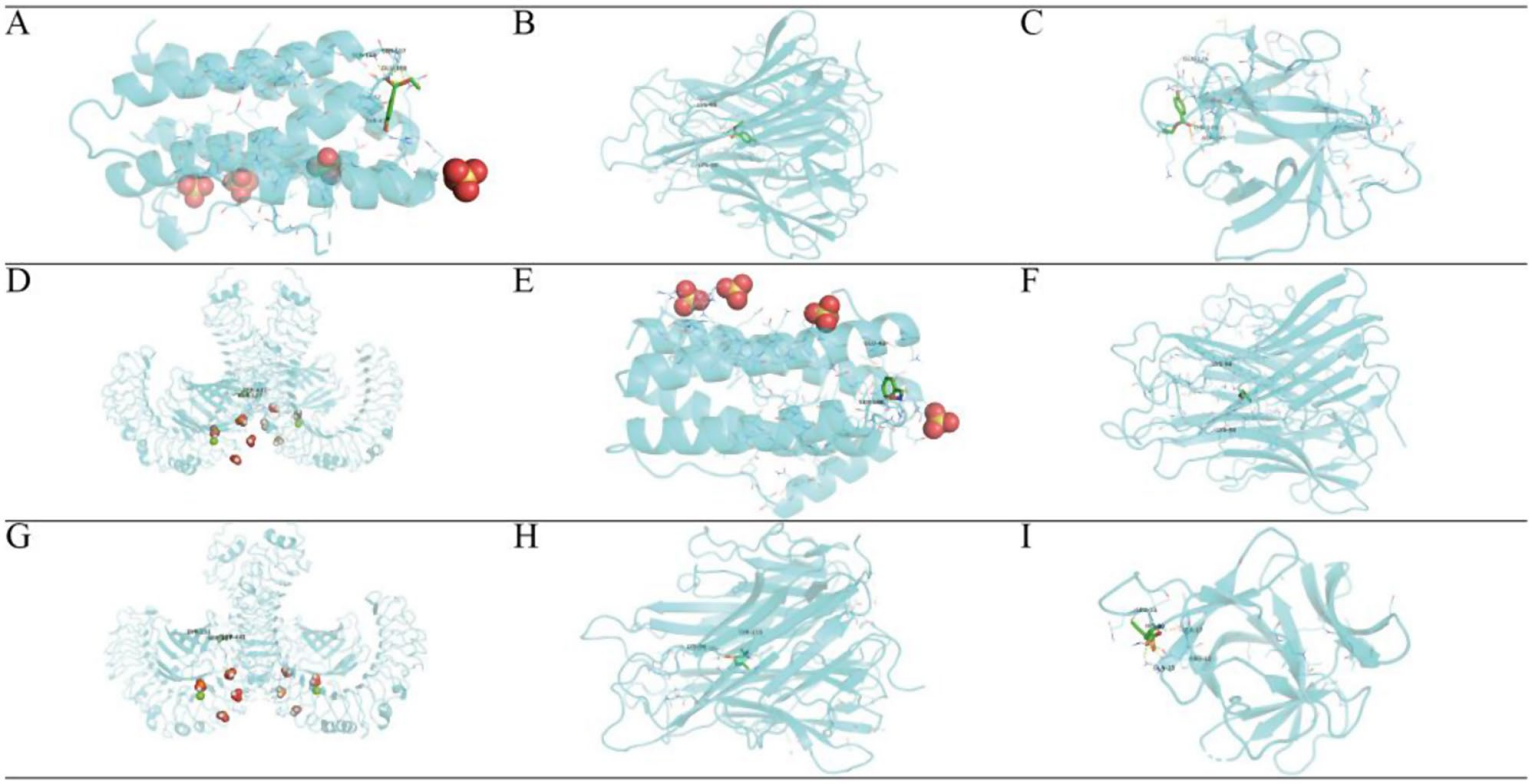
The interactions between protein receptors and ligands. (**A**) Sobrol A binds to IL6. (**B**) Sobrol A binds to TNF. (**C**) Sobrol A binds to IL1B. (**D**) Sobrol A binds to TLR4. (**E**) Benzaldoxime binds to IL6. (**F**) Benzaldoxime binds to TNF. (**G**) Benzaldoxime binds to TLR4. (**H**) (+)-L-Alliin binds to TNF. (**I**) (+)-L-Alliin binds to IL1B

The results of molecular docking of active ingredients to inflammatory factors are shown in Fig. 4A-I. The molecular binding energies of Sobrol A to IL6, TNF and IL1B were all the lowest, indicating that Sobrol A exerts the main anti-inflammatory effect. Among them, Sobrol A had seven binding hydrogen bonds to IL6 with the following binding sites: SER-107, SER-108, GLU-106, GLU-42 and THR-43. In addition, Benzaldoxime and (+)-L-Alliin also showed good molecular binding activity to inflammatory factors.

TLR4 plays an important role in the study of ALD. By molecular docking techniques, we found that Sobrol A, Benzaldoxime and (+)-L-Alliin have strong binding ability to TLR4. Among them, Benzaldoxime has four binding hydrogen bonds to TLR4 with the binding sites: SER-127, SER-441 and TYR-131.

**Fig. 5:**
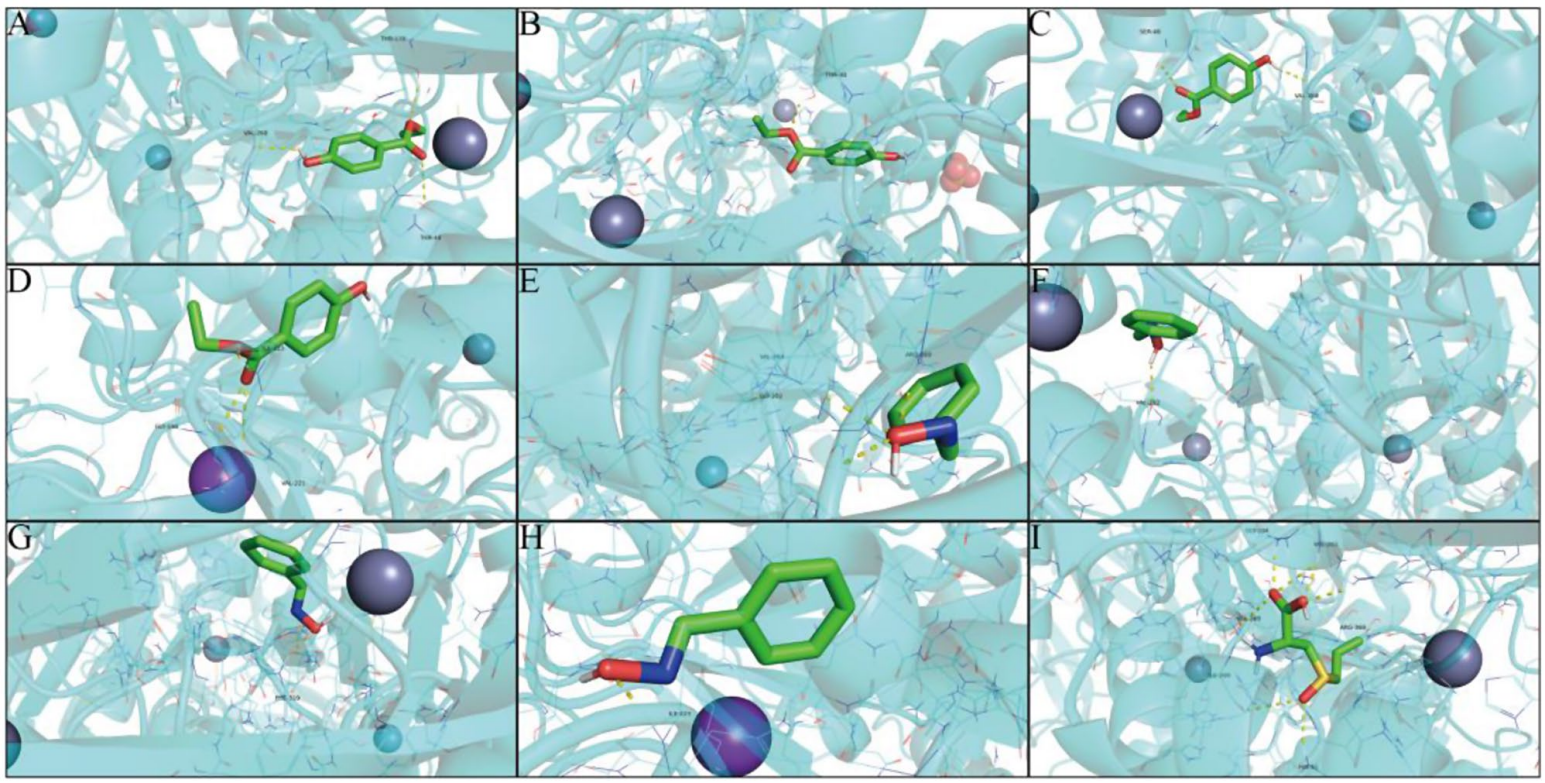
The interactions between protein receptors and ligands. (**A**) Sobrol A binds to ADH1A. (**B**) Sobrol A binds to ADH1B. (**C**) Sobrol A binds to ADH1C. (**D**) Sobrol A binds to ADH5. (**E**) Benzaldoxime binds to ADH1A. (**F**) Benzaldoxime binds to ADH1B. (**G**) Benzaldoxime binds to ADH1C. (**H**) Benzaldoxime binds to ADH5. (**I**) (+)-L-Alliin binds to ADH1A

The results of molecular docking of the active ingredient to alcohol dehydrogenase are shown in Fig. 5A-I. Sobrol A has the best binding stability to ADH1A, ADH1B, ADH1C and ADH5, while (+)-L-Allicin, Benzaldoxime and Allicin also have some binding ability. Of these, (+)-L-Alliin had seven binding hydrogen bonds to ADH1A with the following binding sites: GLY-204, VAL-203, VAL-268, ILE-269, ARG-369 and HIS-51.

**Fig. 6:**
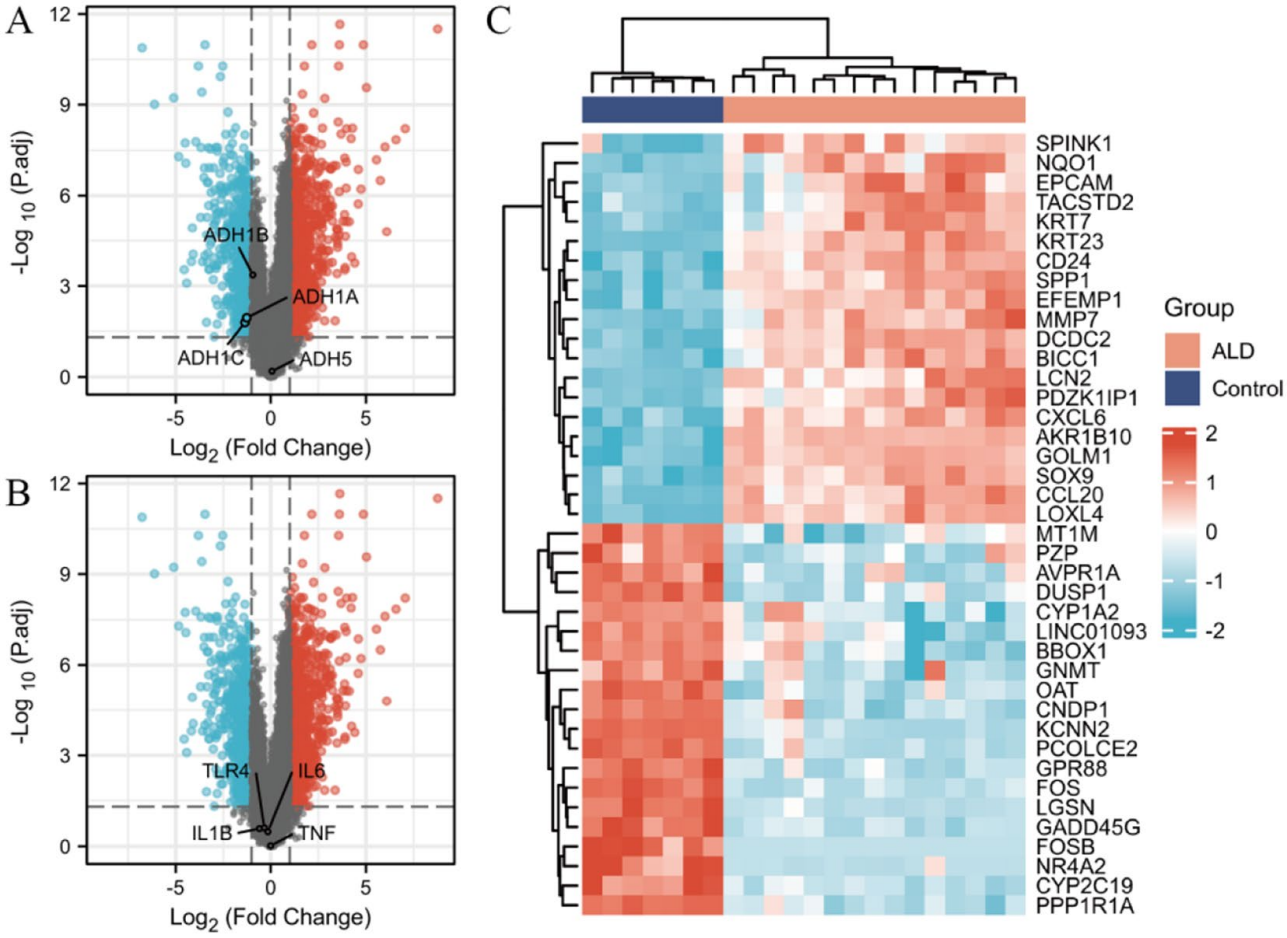
Expression of key genes in human tissues in ALD. (**A**) Expression of alcohol dehydrogenases ADH1A, ADH1B, ADH1C, and ADH5. (**B**) Expression of inflammatory factors TNF, IL1B, IL6 and receptor TLR4. (**C**) Other potential key pathogenic genes in ALD

### Expression status of key genes in ALD in human tissues

To identify key genes associated with ALD, GSE28619 was divided into a reference group and an experimental group. Normal tissue samples served as the reference group, while ALD tissue samples constituted the experimental group. The screening conditions were |log_2_ FC| > 1 and *P* < 0.05. The differential genes in the dataset are shown in Fig. 6. In Fig. 6A, we found that ADH1A and ADH1C were down-regulated in liver tissues of alcoholic hepatitis patients, while ADH1B and ADH5 were not significantly changed. In contrast, there was no significant difference in the expression of inflammatory factors TNF, IL6, IL1B and receptor TLR4 (Fig. 6B). Furthermore, it is possible that these differential genes in Fig. 6C play an important role in the development of alcoholic hepatitis and are the direction of our follow-up study.

### Allicin treatment attenuated pathological changes and ADH1A/ADH1B/ADH1C/ADH5 levels in alcoholic liver disease mice

**Fig. 7:**
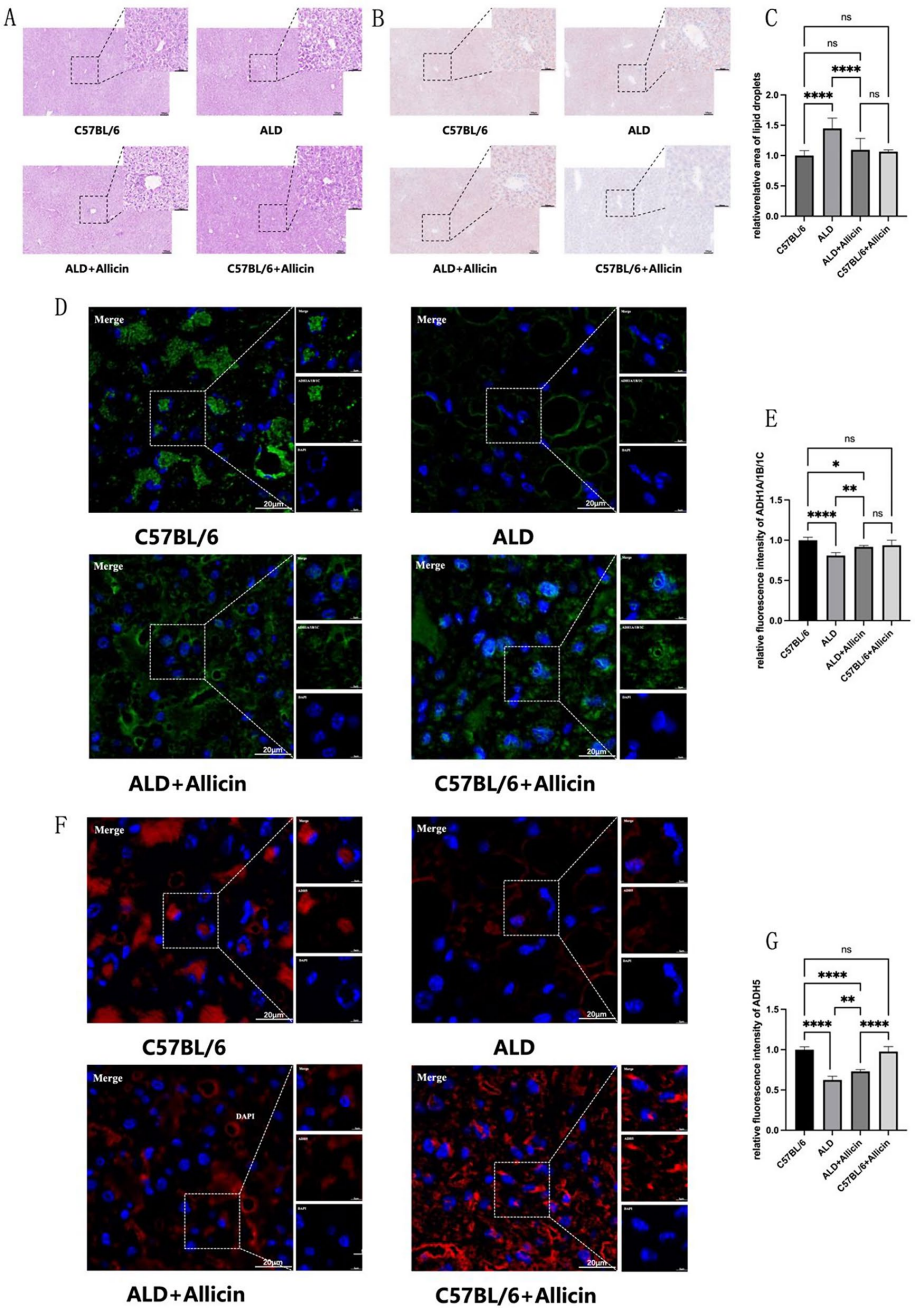
**(A)** Hematoxylin and eosin (HE) staining of mouse liver tissues at 10× and 40× magnifications was performed. **(B)** Oil Red O staining of mouse liver tissues at 10× and 40× magnifications was conducted to assess lipid content. **(C)** Quantification of the positive area in the Oil Red O staining was performed. (**D**,** F)** Liver sections underwent immunostaining using ADH1A/ADH1B/ADH1C, and ADH5 antibodies. **(E**,** G)** Quantification of the relative fluorescence intensity resulting was carried out. (*P < 0.05, **P < 0.01, ***P < 0.001, ****P < 0.0001, indicating statistically significant data between groups; ns indicates no statistical significance)

In Fig. 7, we investigated the histopathological morphology of liver tissues in mice model of alcoholic liver disease. H&E staining revealed that, compared to the C57BL/6 group, ALD group mice exhibited an unclear liver lobule structure, narrowed hepatic sinusoids, disordered arrangement of hepatocyte cords, noticeable fatty vacuoles, and significant inflammatory cell infiltration. Following allicin treatment in the ALD + Allicin group, the lesions in mice were markedly reduced. In the C57BL/6 and C57BL/6 + Allicin groups, the liver lobule structure was normal, hepatocytes arranged in a plate-like structure around the central vein, and occasional fatty degeneration, with no necrosis and minimal inflammatory cell infiltration (Fig. 7A). The liver showed almost no significant pathological changes.

Oil Red O staining, a common pathological examination, aided in assessing the fat content within hepatocytes. In the alcoholic liver disease mouse model, hepatocellular fat vacuoles in the ALD group were prominently red-stained. Further quantitative analysis revealed a significant increase in the positive area ratio of liver cells. Allicin treatment effectively reduced the red-stained area and the positive area ratio of liver cells compared to the ALD group (Fig. 7B and C). Immunofluorescence was utilized to detect the levels of ADH1A/ADH1B/ADH1C, ADH5 in liver tissues. Allicin treatment significantly increased the levels of ADH1A/ADH1B/ADH1C, ADH5 compared to the ALD group mice (Fig. 7D-G).

### Allicin treatment reduced the levels of inflammatory factors and TLR4 expression in alcoholic liver disease model mice

**Fig. 8:**
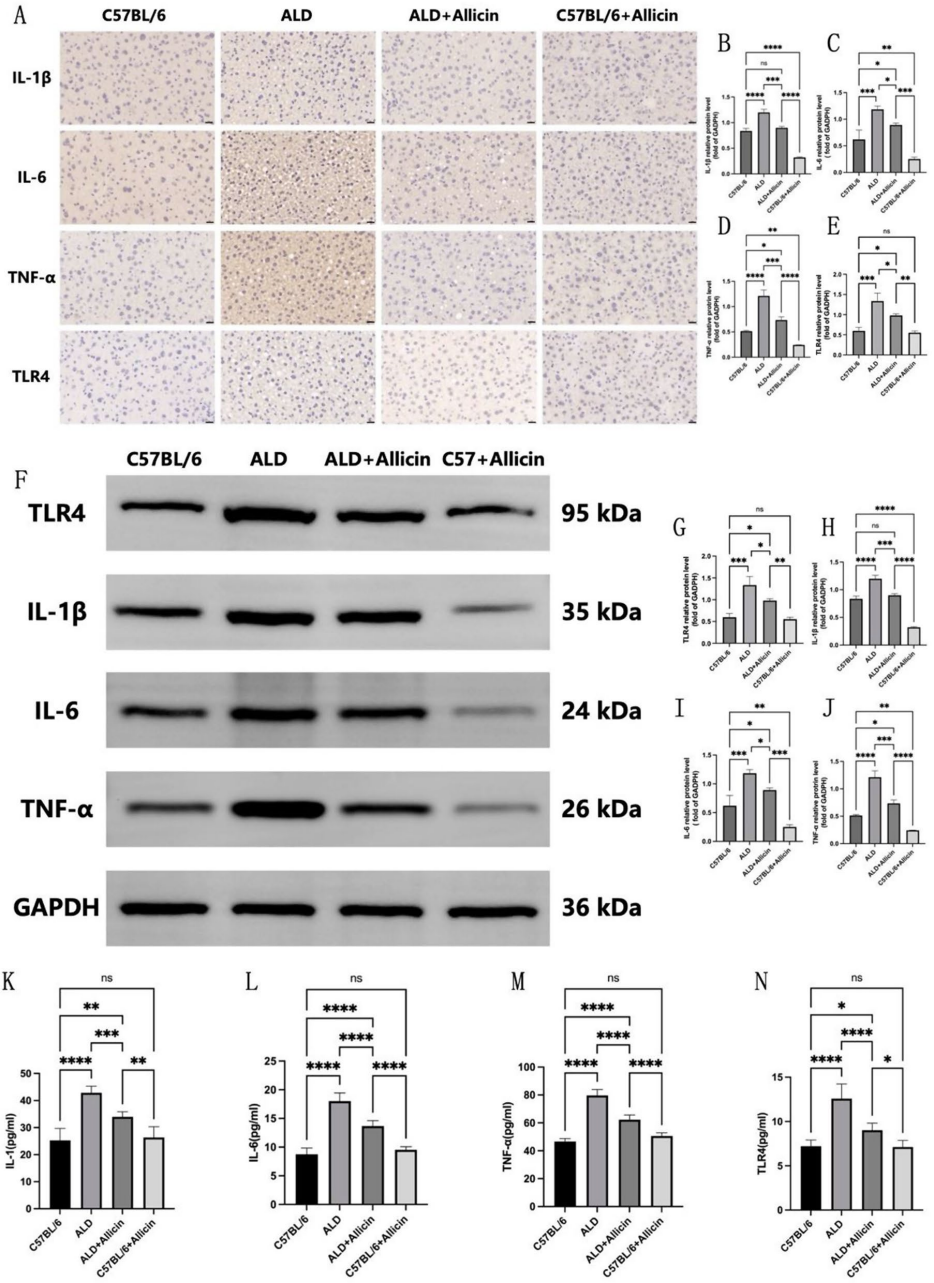
**(A)** Immunohistochemistry (IHC) staining of IL-1β, IL-6, TNF-α and TLR4 in liver of C57BL/6 (*n* = 3), ALD (*n* = 3), ALD + Allicin (*n* = 3), C57BL/6 + Allicin (*n* = 3). (**B**-**E**) The number of IL-1β-, IL-6-, TNF-α-, and TLR4-positive cells in the liver was quantified. (**F**-**J**) TLR4, IL-1β, IL-6 and TNF-α protein expression in the liver of mice in each group (*n* = 3) were examined by Western blotting (WB) **(F)** and quantified by normalizing to GAPDH (**G**-**J**). (**K**-**N**) IL-1β, IL-6, TNF-α and TLR4 levels in plasma of C57BL/6 (*n* = 6), ALD (*n* = 6), ALD + Allicin (*n* = 6), C57BL/6 + Allicin (*n* = 6) were measured by enzyme linked immunosorbent assay (ELISA). (*P < 0.05, **P < 0.01, ***P < 0.001, ****P < 0.0001, indicating statistically significant data between groups; ns indicates no statistical significance)

The impact of allicin on inflammatory factors in mice is illustrated in Fig. 8. Results from WB and immunohistochemistry experiments on liver tissues indicated a significant elevation in the levels of pro-inflammatory factors (TNF-α, IL-6, IL-1β), and TLR4 in the liver of ALD group mice compared to the C57BL/6 group. In contrast, the ALD + Allicin group exhibited significantly reduced levels of IL-1β, IL-6, TNF-α, and TLR4 in the liver compared to the ALD group, as confirmed by both WB and immunohistochemistry experiments (Fig. 8A-J), with statistically significant differences.

Further analysis of allicin’s impact on anti-inflammatory factors and TLF4 expression in mouse serum is presented in Fig. 8K-N. Consistent with tissue expression results, compared to the C57BL/6 group, the serum levels of IL-1, IL-6, TNF-α, and TLR4 were significantly increased in ALD group mice, with statistical significance. After allicin administration, the ALD + Allicin group demonstrated a significant decrease in the levels of IL-1, IL-6, TNF-α, and TLR4 compared to the ALD group, with statistical significance. Notably, the expression levels of inflammatory factors and TLR4 in the C57BL/6 + Allicin group showed no significant difference compared to the C57BL/6 group.

## Discussion

Garlic, an aromatic herb employed in human healthcare through traditional medicines, spices, and various food ingredients [36], serves as a prolific source of novel pharmacologically active compounds [37]. The bulbs of garlic harbor a multitude of phytochemicals, and their extracts and active constituents exhibit a diverse array of biological properties, offering potential protective effects against a spectrum of diseases, including rheumatism, diabetes, cardiovascular conditions, cancer, liver ailments, bronchitis, and tumor growth [36, 38]. Many drugs and antibiotics currently available in the market manifest diverse toxic side effects and withdrawal issues [10, 39]. As a result, there is a growing interest in herbal extracts and pharmacologically active compounds derived from plant species utilized in traditional medicine.

In our investigation, we systematically examined 26 active molecular components of garlic, including Allicin, Alliin, 2-Isopropyl-1,3-dioxolane, Dimerthylthiophen, DATs, SobrolA, 2-ethyl-1-3-dithi, 3-prop-1 ene, and others for clarity and conciseness. Some of these components, including Allicin, have demonstrated hepatoprotective effects. Allicin, for instance, exhibits anti-inflammatory properties by suppressing the expression of LPS, CD14, TLR4, TNF-α, IL-1β, and IL-6, thereby ameliorating the lipopolysaccharide (LPS)-CD14-toll-like receptor 4 (TLR4)-induced inflammatory pathway in the liver [40, 41]. Certain compounds in garlic, such as allin, allyl cysteine, diallyl disulfide, and allyl disulfide, act as scavengers of superoxide anion radicals, mitigating lipid peroxidation and hepatocyte damage [42–44]. Furthermore, treatment of cells (Caco-2 and THP-1) with propyl propane thiosulfonate, a component of garlic, resulted in a noteworthy reduction in the synthesis of pro-inflammatory mediators (IL-1β, TNF-a, IL-8, IL-17, and ICAM-1) and suppressed inflammatory immune responses by down-regulating mitogen-activated protein kinase signaling pathways (MAPKs) [45]. Animal studies have corroborated that the organosulfide content of garlic can diminish alcohol-related liver enzymes and enhance the expression of hepatic antioxidant enzymes, thereby serving as a hepatoprotective agent [17, 46, 47]. Consequently, our results underscore that the active chemical components in garlic may offer therapeutic benefits in the context of ALD.

In conjunction with prior studies, we predicted the major active ingredients and targets of action in garlic, identifying a total of 83 potential therapeutic targets through the intersection of drug and disease targets. The subsequent GO and KEGG pathway enrichment analysis of these 83 target genes revealed a total of 1034 biological functions and 107 signaling pathways. Some of these gene functions and pathways were directly implicated in the development and progression of ALD, suggesting potential mechanisms through which garlic may treat ALD. Numerous signaling pathways closely associated with ALD include Drug metabolism-cytochrome P450 [48, 49] and Metabolism of xenobiotics by cytochrome P450 pathways [50, 51]. Cytochrome P450, an alcohol-metabolizing enzyme, generates reactive oxygen species during alcohol metabolism, leading to heightened oxidative stress, nitrosative stress, and lipid peroxidation. Elevated lipid peroxidation and protein oxidation are crucial features of alcoholic liver injury. In the Chemical carcinogenesis-DNA adducts pathway, the alcohol metabolite acetaldehyde binds to proteins and DNA, forming protein adducts and carcinogenic DNA adducts. Long-term alcohol consumption initiates liver damage as the primary stage, followed by cirrhosis, significantly escalating the risk of chemical carcinogenesis [52, 53].

Furthermore, the most crucial signaling pathway associated with Alcoholic Liver Disease (ALD) is presented in Supplementary Fig. 1, featuring target genes such as IL6, IL1B, TNF, TLR4 receptor, and alcohol dehydrogenases (ADH1A, ADH1B, ADH1C, and ADH5). Chronic exposure to ethanol sensitizes Kupffer cells to lipopolysaccharide activation via Toll-like receptors (e.g. TLR4), resulting in enhanced production of pro-inflammatory factors such as interleukin-1 (IL-1), tumor necrosis factor-alpha, and interleukin-6. This cascade leads to hepatocyte dysfunction, necrosis, apoptosis, and the generation of extracellular matrix proteins, culminating in fibrosis/cirrhosis [54, 55]. Recent findings indicate that the neutralizing effect of IL-1 receptor antagonists on IL-1 is effective in preventing liver injury in a mouse model of ALD [56, 57]. However, our analysis of the GSE28691 database showed that ADH1A and ADH1C were downregulated in liver tissues of patients with alcoholic hepatitis, while ADH1B and ADH5 showed no significant changes. There was no significant difference in the expression of TNF, IL6, IL1B, and TLR4 between the two groups. These results are inconsistent with our previous conclusions from network pharmacology and molecular docking, as well as those from the literature. Therefore, after excluding possible errors in analytical methods, we designed experiments and verified the results using WB, immunohistochemistry, and ELISA. Compared with C57BL/6 mice, ALD mice showed increased expression of inflammatory factors (IL-1β, IL-6, TNF-α) and TLR4 in liver tissues, and similar results were observed in plasma. Immunofluorescence staining confirmed that the levels of ADH1A, ADH1B, ADH1C, and ADH5 in liver tissues of C57BL/6 mice were significantly higher than those in the ALD group. The limitations of using only a single GEO dataset may affect the generalizability and interpretation of the results, indicating the importance of employing a broader set of datasets in future studies. This also highlights the crucial role of experimental verification in scientific research.

The primary pathway for the oxidative metabolism of ethanol in the liver involves the oxidative metabolism of alcohol dehydrogenase (ADH) [58]. This metabolism produces acetaldehyde, a highly toxic by-product known to cause liver tissue damage and potentially lead to alcohol dependence [59]. Notably, ADH expression was found to be downregulated in the liver of patients with Alcoholic Liver Disease (ALD), suggesting a diminished ability of the liver to detoxify ethanol [34, 60]. Consequently, we hypothesize that garlic may enhance ethanol metabolism by increasing ADH activity, acting as an inhibitor of ALD progression. Immunofluorescence assays revealed decreased levels of alcohol dehydrogenase (ADH1A/ADH1B/ADH1C and ADH5) in ALD mice compared with control mice.

Interestingly, these targets also play a significant role in vascular disease. Atherosclerosis (AS) is a chronic inflammatory disease where the release of pro-inflammatory cytokines (e.g. IL1β, IL6, and TNF) promotes its development [61–63]. Peroxisome proliferator-activated receptor-gamma (PPARG) is a key factor in foam cell formation and lipid uptake in AS [64, 65]. Additionally, matrix metalloproteinase 9 (MMP9) is a crucial protein hydrolase involved in degrading various extracellular components of the aortic wall and is implicated in the pathogenesis of abdominal aortic aneurysms [66, 67].

Ten core genes associated with garlic for ALD treatment, including IL6, TNF, AKT1, IL1B, HIF1A, PTGS2, TLR4, PPARG, HSP90AA1, and PPARA, were identified using the cytohubba algorithm and alcoholic disease-related signaling pathway screening. Molecular docking techniques were employed to predict the binding strength of garlic active ingredients to core target genes. Among these, Sobrol A, (+)-L-Alliin, and benzaldoxime demonstrated robust binding to four core anti-inflammatory genes (IL6, IL1β, TNF, and TLR4), indicating their potential anti-inflammatory and antioxidant effects. Additionally, alcohol dehydrogenase-related genes (ADH) exhibited stable binding to three active ingredients—Sobrol A, (+)-L-Alliin, and Allicin, underscoring their relevance to alcohol metabolizing enzymes, all with docking intensities <-4.2 kcal/mol. Our molecular docking validation results further underscore that these active ingredients targeting core genes are pivotal for garlic’s efficacy in ALD treatment. These findings were experimentally verified, particularly in the case of Allicin treatment in alcoholic liver disease mice, which demonstrated elevated ADH activity and reduced inflammatory factors and TLR4 expression compared with ALD mice. Negative controls were included, revealing lower levels of inflammatory factors and TLR4 in the liver tissues of the C57BL/6 + Allicin group compared to the C57BL/6 group, with no significant difference in plasma levels. Compounds such as Sobrol A, (+)-L-Alliin, Benzaldoxime, and Allicin exhibit notable affinity with key ALD-associated genes, including IL6, IL1β, TNF, the TLR4 receptor, and alcohol dehydrogenase genes (ADH1A, ADH1B, ADH1C, and ADH5). These compounds, Sobrol A, (+)-L-Alliin, Benzaldoxime, and Allicin, emerge as particularly promising natural drug candidates for the treatment of ALD.

## Conclusion

In summary, this study employed network pharmacology and molecular docking techniques to predict the crucial active compounds and key targets of garlic in treating ALD, speculating on potential mechanisms of action from various pathways and perspectives. Several results obtained through network pharmacology and molecular docking were experimentally verified. The findings suggest that garlic could impede disease progression by mitigating the inflammatory response and enhancing ethanol metabolism. Notably, SobrolA, (+)-L-Alliin, Benzaldoxime, and Allicin exhibited the most targets and displayed high activity, making them potential candidates for future pharmacological interventions in ALD treatment.

### Electronic supplementary material

Below is the link to the electronic supplementary material.

Supplementary Material 1

## Data Availability

The expression profiles of clinical samples were available in the GEO database. The original contributions presented in the study are included in the article/supplementary material. Further inquiries can be directed to the corresponding authors.
