# Supplementary material for: Exploring the therapeutic potential of garlic in alcoholic liver disease: a network pharmacology and experimental validation study
Source: Genes Nutr. 2024 Jul 23;19:13. doi: 10.1186/s12263-024-00748-3 (PMC11267778; doi:10.1186/s12263-024-00748-3)
Supplement: Supplementary file 1 — Supplementary Material 1 [file 12263_2024_748_MOESM1_ESM.docx]

Supplementary Material

## Supplementary Figures


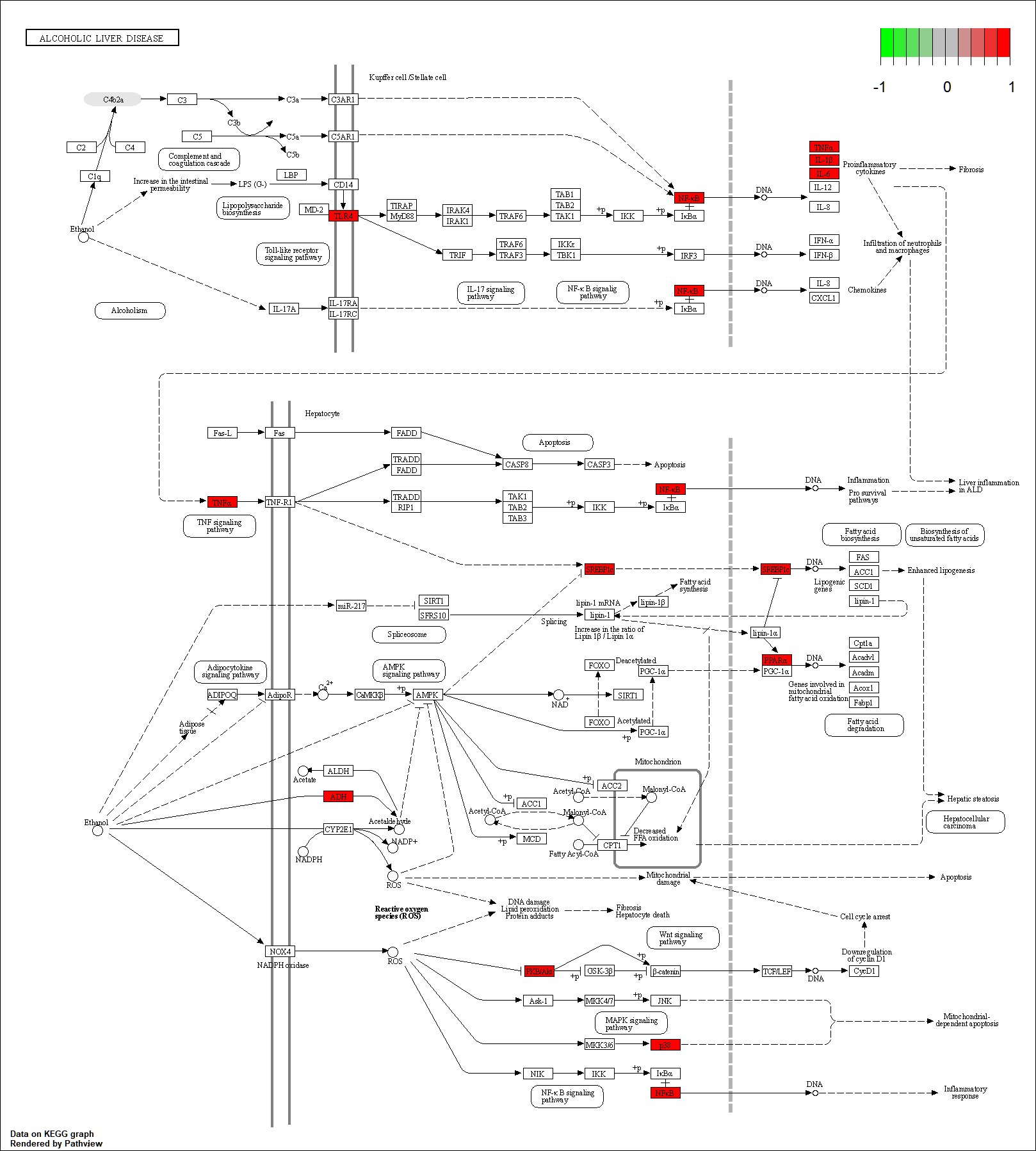


**Supplementary Figure 1.** The Alcoholic Liver Diseases signaling pathway. Arrows indicate activation effects; the red targets are the pathways we focused on in this study.
